# A Novel Catalytic Ceramic Conversion Treatment of Zr702 to Combat Wear

**DOI:** 10.3390/ma16051763

**Published:** 2023-02-21

**Authors:** Xinhe Xiong, Xiaoying Li, James Alexander, Zhenxue Zhang, Hanshan Dong

**Affiliations:** School of Metallurgy and Materials, University of Birmingham, Birmingham B15 2TT, UK

**Keywords:** ceramic conversion treatment, wear resistance of Zr702, catalytic films

## Abstract

Zr and its alloys are widely used in multiple areas, including the nuclear and medical fields. Previous studies indicate that a ceramic conversion treatment (C2T) of Zr-based alloys can address the issues of low hardness, high friction, and poor wear resistance of Zr based alloys. This paper introduced a novel catalytic ceramic conversion treatment (C3T) to Zr702 by pre-depositing a catalytic film (such as silver, gold, platinum, etc.) before the ceramic conversion treatment, which efficiently promoted the C2T process, in terms of reduced treatment times, with a thick, good quality, surface ceramic layer. The formed ceramic layer significantly improved the surface hardness and tribological properties of Zr702 alloy. Compared with conventional C2T, the C3T technique provided two orders of magnitude reduction of wear factor and reduced the coefficient of friction from 0.65 to <0.25. Among the C3T samples, the C3TAg and the C3TAu samples have the highest wear resistance and lowest CoF, mainly due to the self-lubricant formation during the wear processes.

## 1. Introduction

Zirconium (Zr) and its alloys have found applications in the nuclear, chemical, and medical industries due to their excellent corrosion resistance, low coefficient of thermal expansion (CTE), high melting points, non-toxicity, and relatively good biocompatibility. However, Zr and its alloys are characterised by low hardness, high friction, and poor wear resistance, which have limited the wider applications of Zr and its alloys in the above-mentioned fields [1,2,3,4]. Some surface engineering methods, such as vapour deposition (PVD and CVD), ion implantation, laser treatment, etc. [5,6,7,8], have been explored to improve the tribological properties of Zr and its alloys. However, laser treatment ends up with very rough surfaces, and costly post-machining is essential [9]. Other treatments are unable to produce high load bearing capacity. This is because the ion implanted layer is very thin (within several hundreds of nanometres) [10] and the egg-shell effect was frequently observed for PVD and CVD hard ceramic coatings on soft Zr and its alloys under medium to high load [11]. Some researchers investigated nitriding of Zr alloys [12], but distortion at high treatment temperature and very thin treated layers are the major technical barriers.

Therefore, a special surface ceramic conversion treatment (CCT) based on thermal oxidation has been developed [13]. Under optimal treatment conditions, a dense ceramic surface layer can be converted from the Zr matrix, which significantly improves the wear and corrosion resistance of the Zr alloys. However, a relatively long processing time (50–200 h) is needed to obtain a reasonably thick ceramic layer for tribological applications [2], which is excessively energy consuming and inefficient for industrial production.

Recently, Zhang et al. [14] found that a pre-deposited thin gold layer could greatly speed up the CCT process of Ti6Al4V. The treatment time can be greatly reduced from 60–80 h to only 8–10 h at 620 °C to generate a compact and hard surface oxide layer with excellent tribological properties. Moreover, Alexander et al. [15] discovered that a co-deposited layer of Ag/Pd on titanium fixation pins accelerated the formation of a good quality surface oxide layer on their complex surfaces in 5 h, which well-outperformed the untreated pins, in terms of reduced drilling force and antibacterial efficacy.

As zirconium is in the same periodic group as titanium and possesses the same crystal structure with similar chemical properties and industry applications, it is naturally interesting to investigate whether pre-deposition elements have the same accelerating effect on forming good quality oxide layers on zirconium and its alloys for improved tribological performance. However, there has been no report in the literature, and there is a knowledge gap on applying such techniques on zirconium-based alloys, and the optimal conditions (i.e., most effective pre-deposited layer, treatment temperatures, and treatment duration) for the C3T technique are yet to be found. Therefore, it is scientifically interesting and technologically important to explore the feasibility for accelerating the CCT process for Zr and its alloys by pre-deposited selected elements to obtain a high-quality oxide layer for combating the wear of Zr and its alloys. The purposes of this paper are: (a) to study the effect of pre-deposited metal films on the ceramic conversion treatment of zirconium alloys; (b) to study the microstructure and evaluate tribological performance of the converted surface oxide layers, and (c) to discuss the possible catalytic effects on the fast growth of the oxide layer on zirconium alloys.

## 2. Materials and Methodologies

### 2.1. Materials and Sample Preparation

Commercial grade Zr702 was used as substrate material, and the chemical composition from the supplier’s specification is listed in Table 1. The specimens were cut from Zr702 bar of 24 mm diameter using a precision cut-off machine (Struers, Rotherham, UK, Accutom-50) to coupons of 4.5 mm in thickness. The samples were ground with abrasive SiC papers from grades 240 to 1200 and cleaned with soapy water and acetone in an ultrasonic bath between every grinding step.

Catalytic ceramic conversion (C3T)-treated coupon samples were cut perpendicular to the treated surface and mounted with conductive Bakelite for cross-sectional layer structure observation. The mounted samples were ground from 240 to 2500 with abrasive SiC papers and then polished with a MD-Mol polishing cloth with 3-micron diamond suspension applied. The final polishing was completed using activated collodial silica on an MD-chem polishing cloth.

### 2.2. Pre-Deposition of Thin Metal Films

A thin layer of silver (Ag), vanadium (V), or palladium (Pd) was deposited on the samples prepared via physical vapour deposition, using closed-field unbalanced magnetron sputtering ion plating equipment (Teer Coating Ltd., Worcestershire, UK) with a bias current 0.2 A (for Ag, V, and Pd targets) for 180 s. The gold coating was obtained using a sputter coater (Emscope SC 500, Hertfordshire, UK) with a current of 25 mA for 180 s. The thickness of the pre-deposition layer was controlled to be in the range of 300–350 nm.

### 2.3. Ceramic Conversion Treatment

The Ag, V, Pd, and Au pre-deposited and uncoated samples were ceramic conversion treated in a muffle furnace (Elite Thermal Systems Limited, Leicestershire, UK) at set temperatures of 450, 500, 550 and 600 °C for 10 h. As introduced in Section 1, the pre-deposition metal elements are used to accelerate the surface oxide layer formation (i.e., catalytic effect); therefore, it is coded as C3T (catalytic ceramic conversion treatment) for the samples with pre-deposition of a metal film. The heating ramp rate was 8 °C/min, and when the treatment was finished, the samples were cooled down within the furnace. The treatment conditions and the designated sample code for all samples are summarised in Table 2. The original untreated samples, named as Unt, and the sample treated with no pre-deposition of metal film, named as C2T, are in the table for comparison.

### 2.4. Microstructure Characterisation and Property Evaluation

The surface morphology and cross-section microstructures and the corresponding chemical compositions of all treated samples were studied with SEMs (FEI Quanta 3D FEG FIB-SEM, Hillsboro, OR, USA, and Hitachi TM-3030, Tokyo, Japan) equipped with energy dispersive X-ray spectroscopy (EDX). The thickness of formed surface oxide layer was measured from the SEM cross-sectional images, and the average value of five measurements for each sample is reported. X-ray diffraction (XRD) scans were carried out on all the samples using a Proto AXRD (Wrocław, Poland) with a Cu target (Kα = 0.154 nm) at a scan rate of 0.03°/s, and the collected XRD data were analysed using HighScore software (Malvern Panalytical Ltd., Malvern, UK) with PDF [16] for phase identification.

The surface roughness, R_a_ (arithmetic average roughness) and R_t_ (data points range), was measured before and after the treatments using an Ambios XP-200 stylus profilometer, and the average values of three measurements of 600 μm were reported. A Mitutoyo MVK-H1 Vickers microhardness tester (Japan) was used to measure the surface hardness of the samples with a load of 50 g and 100 g (a few were at 25 g for comparison), and the average value of five indents are reported.

A phoenix TE-79 multi-axis tribology (UK) machine was used to evaluate the friction behaviour of the treated samples, compared with the untreated ones. The dynamic coefficient of friction for treated and untreated samples were measured with a tungsten carbide ball (8 mm in diameter) as the counterpart, under a normal load of 20 N, a sliding stroke of 5 mm, and a sliding speed of 5 mm/s for 1000 cycles. Wear tests were performed on a homemade reciprocating sliding wear tester with a tungsten carbide ball counterpart (12 mm in diameter), under a normal load of 21 N, for 3600 cycles with sliding stroke of 5 mm. Wear tracks were probed by the Ambios XP-200 stylus profilometer, three 2D scans across the wear tracks perpendicular to the sliding direction were measured, and the average wear area, volume, and rate were calculated. Further SEM observation and EDX analysis on the wear tracks were taken to investigate the wear features and mechanisms involved.

## 3. Results and Discussion

### 3.1. Catalytic Effect as a Function of Treatment Temperature

**Figure 1:**
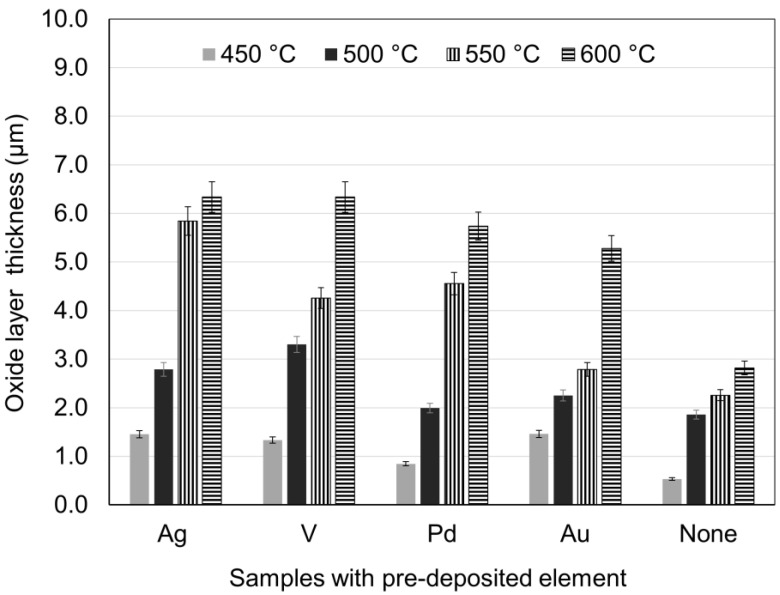
Surface oxide layer thickness of C2T 10 h processed samples with and without pre-deposited metal elements at different temperatures.

The selected treatment temperatures were between 450–600 °C to avoid degradation of bulk properties and break away of the formed oxide layer at high temperatures [17]. Figure 1 shows the surface layer thickness of the samples with pre-deposited elements as a function of different C2T temperatures for a constant treatment time of 10 h, compared with no pre-deposition samples. It can be seen that the samples with pre-deposited elements (C3T) formed a thicker surface oxide layer than the samples without pre-deposition of metal films (C2T), and the extent of the thickening effect is both element- and treatment temperature-dependent.

It is clear that, when treated under the same temperature, the pre-deposition of these metal films can promote the formation of the surface oxide layers, showing a catalytic effect. When treated below 550 °C, the thickness of the formed surface oxide layers for the C3T samples are relatively thin and most of them are less than 3 µm (except for V pre-deposited sample, which is slightly over 3 µm). When samples were treated at 550 °C, Ag, Pd, and V pre-deposition effectively increased surface oxide layer thickness to 5.8, 4.7, and 4.2 µm, respectively, which is 2.5, 2.0, and 1.8 times the oxide layer thickness of C2T samples. The Au pre-deposited samples showed relatively less increase in the thickness of the surface oxide layer, as compared with the other pre-deposited samples when treated at 500 and 550 °C. When the treatment temperature increased to 600 °C, the pre-deposited elements increased the surface layer thicknesses to a larger extent, about 5.3–6.3 µm, which is a 96–133% increase of the surface layer thickness than the C2T treated samples.

Although the microstructures and tribological properties were investigated for all the treated samples, only the 600 °C 10 h treated samples are reported in the following sections, considering the load bearing capacity requirement for most demanding industry tribological applications.

### 3.2. Surface Morphology and Roughness

**Figure 2:**
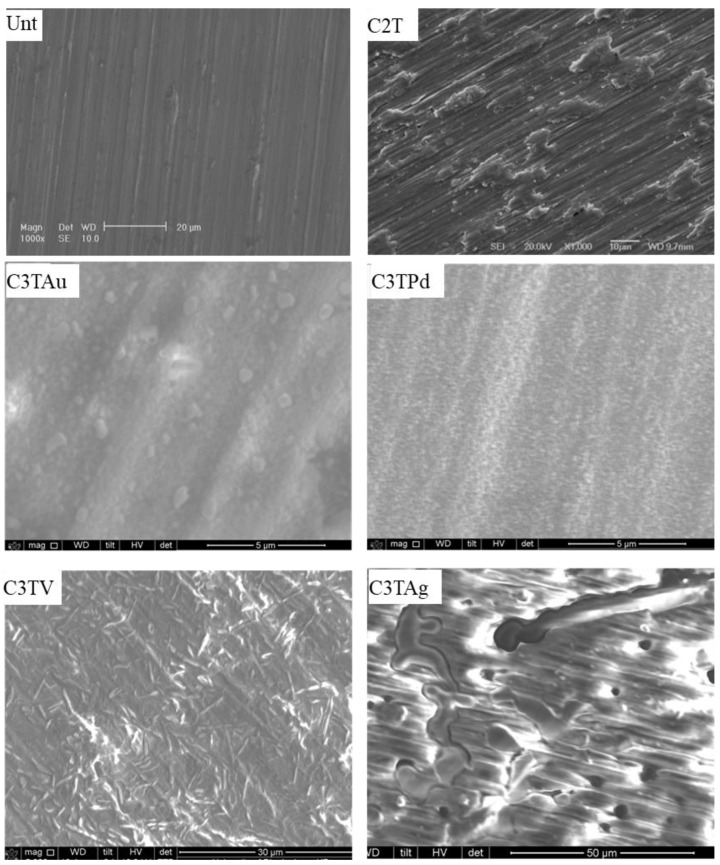
SEM images showing surface morphology of the samples with pre-deposited elements, as shown in the figure, after C3T at 600 °C for 10 h, compared with the untreated sample (Unt) and the sample treated with no pre-deposition layer (C2T).

As shown in Figure 2, the Unt sample surface is covered by grinding traces, while all the treated surfaces revealed characteristic features related to the pre-deposition conditions. For the C2T sample, patches of lumps presented on the surface and the original grinding traces are still visible. The surface of C3TAu sample is covered with equal-axial fine particles with a diameter of 200–400 nm and a few big particles in the diameters of 0.8–1.1 μm. Extremely fine particles with sizes of 150 nm in diameter are evenly presented on the surface of the C3TPd sample. For the C3TV sample, multi-directional needles in the length of 5 µm formed on the surface, which could be the vanadium oxide identified in the XRD analysis (for details, see Section 4.2). Deposited silver particles were agglomerated to columnar-dendrite-like grains with lengths of 50 and widths of 10 µm after the C2T treatment.

**Figure 3:**
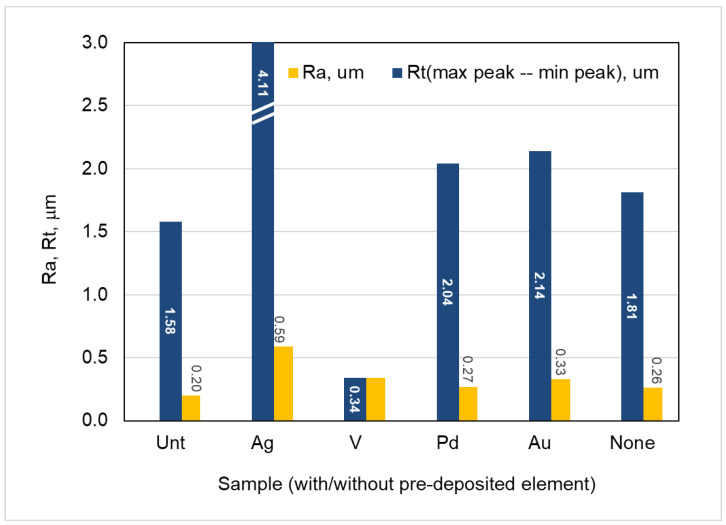
The average Ra and Rt values of the samples after ceramic conversion treatment at temperature 600 °C for 10 h, with and without pre-deposition element, as indicated, compared with untreated substrate sample.

The surface roughness values, in terms of R_a_ (arithmetic average roughness) and R_t_ (maximum and minimum data points range), of the treated samples are presented in Figure 3, together with the untreated sample for comparison. It can be seen that all the treated samples showed increased Ra values, as compared with the Unt sample. It is also revealed that, except for C3TAg, all other C3T samples are only slightly rougher than the C2T sample.

**Figure 4:**
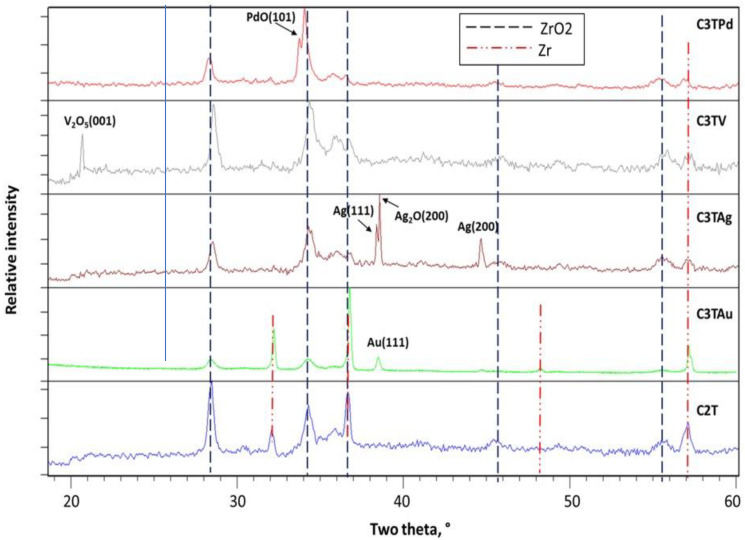
XRD analysis of the samples after the C2T and the C3T process, samples treated at temperature 600 °C for 10 h.

Big contrasts in Rt values, 4.11 and 0.34 mm, were observed for the C3TAg and C3TV samples, respectively. It implies that the observed columnar-dendrite-like grains (Figure 2) on the C3TAg sample may have protruded from the surface of the samples. While, for the C3TV sample, the observed needle-shaped vanadium oxides are finely dispersed on the surface and filled the valleys of the grinding grooves (Figure 2), as the Rt of 0.34 is only one fifth of the Rt for C2T (1.81, Figure 3). The C3TPd and C3TAu samples showed slightly increased Rt values, as compared with that for the C2T sample, which might be related to the small particle sizes of the PdO (identified in XRD analysis, see Figure 4) and gold on the surfaces of the samples (Figure 2).

### 3.3. Surface Layer Structure

**Figure 5:**
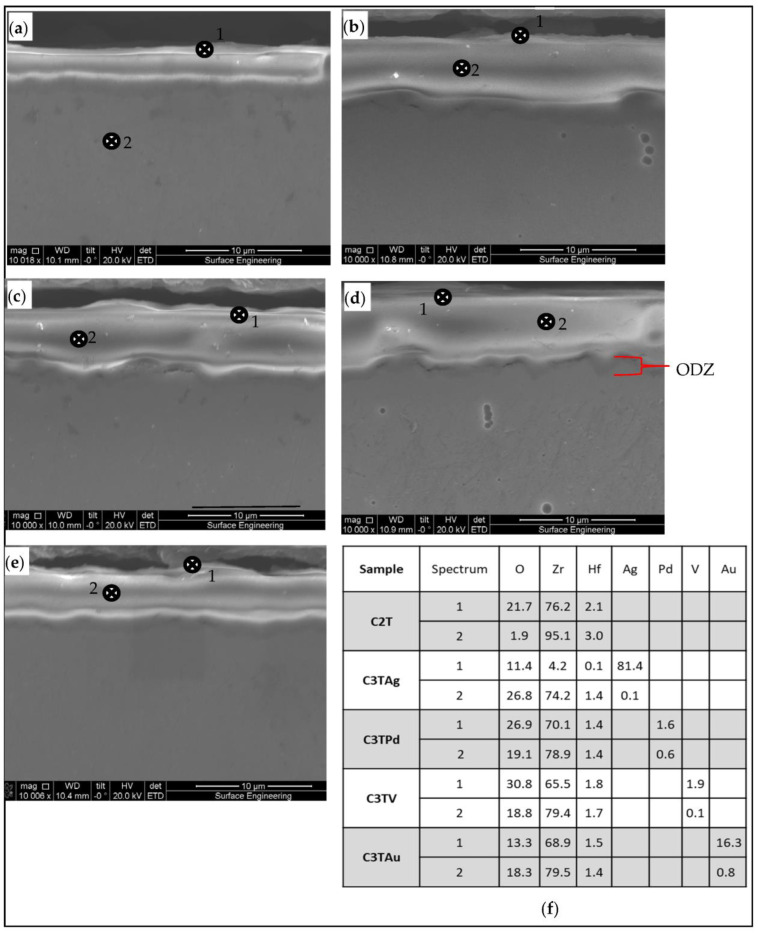
The SEM images of the surface layer structure for samples of (**a**) C2T; (**b**) C3TAg; (**c**) C3TPd; (**d**) C3TV; (**e**) C3Tau; and (**f**) EDX analysis of (**a**–**e**) samples from position 1 and 2, as denoted, compositions are in wt.%.

The XRD patterns of the C2T and C3T samples after 600 °C 10 h treatments are shown in Figure 4. Characteristic peaks of m-ZrO_2_, marked with dash lines in Figure 4 at 2θ positions of 24°, 28.2°, 34.3°, etc., were identified for all the samples, while the α-Zr peaks, marked with double dot dash lines, were detected from the samples with thin surface layers, such as C2T and C3TAu samples. The C3TV sample showed the formation of a vanadium pentoxide (V_2_O_5_) phase, as evidenced by an apparent peak of (001) at 2θ 20.7°. A peak around 2θ positions of 34° was identified as (101) of PdO phase for the C3TPd sample. Two phases related to Ag_2_O and pure silver were identified for the C3TAg sample, as indexed in Figure 4. The EDX analysis on the dendrite-like grains revealed a O/Ag ratio of 1/8 (see Figure 5f), which may indicate that these dendrite-like grains would be composed of Ag and Ag_2_O phases. For the C3TAu sample, a (111) peak of fcc gold phase at 2θ of 38.2° was identified.

Cross-sectional SEM images of 600 °C 10 h treated samples are shown in Figure 5. It can be seen that all C3T samples developed a thicker surface layer than the C2T sample, and their interfaces between the oxide layer and the substrate revealed a wave feature. An oxygen diffusion zone (ODZ) was observed for all treated samples underneath the oxide layer, as exemplified in Figure 5d. The EDX analysis at the outermost surfaces (except for C2T, but with a position in substrate) and within the oxide layers are shown in Figure 5f. It reveals that, after the treatment, the content of pre-deposited element, Pd and V, is moderately higher on the outermost surface than in the oxide layer. While for pre-deposited element of Ag and Au, EDX analysis detected a higher content of 81.4 and 16.3 wt.% at the outermost surface, respectively, which indicated the agglomeration of Ag and Au on the surface during the C3T process, as shown in Figure 2, as dendrite-like grains for C3TAg and granular grains for C3TAu samples.

### 3.4. Hardness and CoF

**Figure 6:**
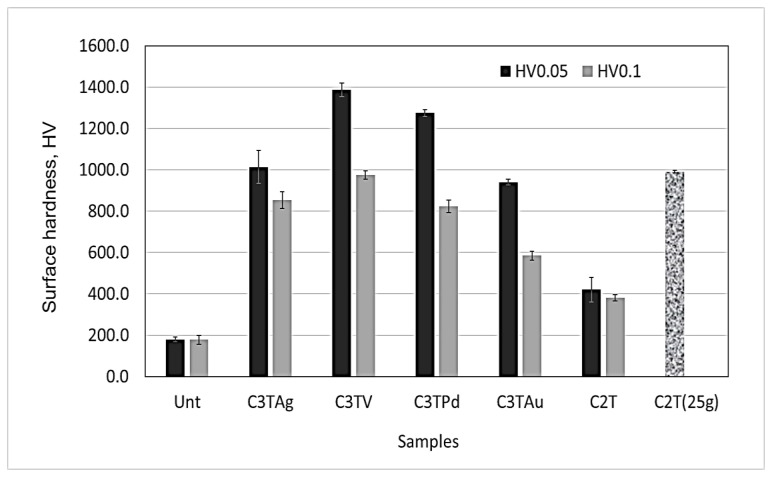
Surface hardness of C2T- and C3T-treated (600 °C, 10 h) samples, in comparison with untreated sample.

Figure 6 plots the surface Vickers hardness of 600 °C/10 h-treated samples, measured under the loads of 0.1, 0.05, and 0.025 kg, compared with the untreated sample. The results show that the ceramic conversion treatments produced a surface hardened layer with a surface hardness between 950 to 1400 HV. The low hardness values shown in Figure 6 are the reflection of the surface layer thickness and the load applied for the measurements. It can be seen that the thinner the surface layer formed, the lower the surface hardness measured with heavier loads, such as 400 HV0.05–0.1 for C2T sample with a surface layer less than 4 µm, and 580 HV0.1 for the C3TAu sample with a surface layer less than 5.5 µm. Further reducing the load to 0.025 kg, a high value of 1000 HV0.025 was gained for the C2T sample. C3TV and C3TPd samples show extra surface hardness of 1380 and 1280 HV0.05, respectively, denoting the hardness contribution from the oxide of V_2_O_5_ and PdO formed on the surfaces (Figure 2 and Figure 4).

**Figure 7:**
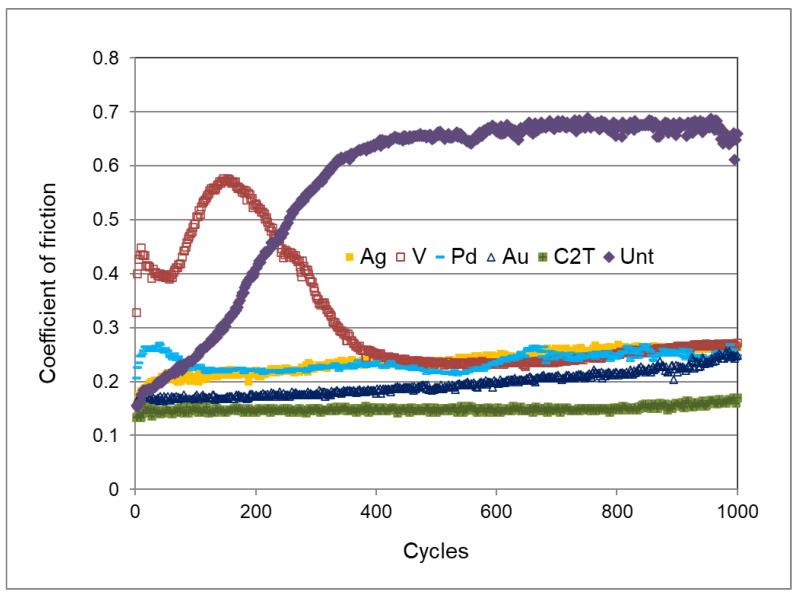
Dynamic Coefficient of Friction (CoF) curves for all C3T samples under the normal load of 20 N, compared with the C2T and Unt samples.

The dynamic curves of the coefficient of friction (CoF), with the WC ball as the counterpart at a load of 20 N for the Unt, C2T, and C3T samples, are presented in Figure 7. The CoF of the untreated Zr702 sample was around 0.15 at the beginning, gradually increased to 0.68 after 400 cycles, and remained steady within the test duration. All ceramic conversion treated samples showed a significantly reduced CoF, to a 0.15–0.27 range, although C3TPd and C3TV showed high values within the 100 and 450 cycles, respectively. This phenomenon could be caused by the high surface hardness of these samples, which will be discussed in Section 3.5.

### 3.5. Wear Resistance

**Figure 8:**
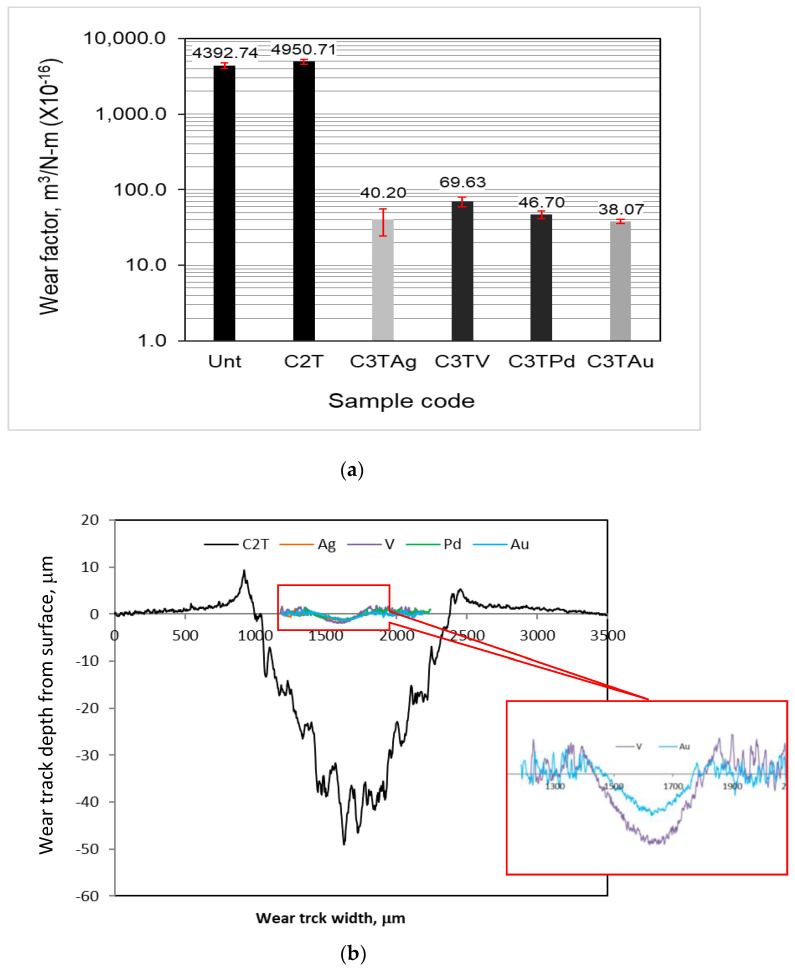
(**a**) Wear factors and (**b**) 2D wear track profiles of untreated and 600 °C10 h treated samples with and without pre-deposition layers (inserted details of the enlarged wear tracks of C3TV and C3TAu samples).

Wear factors of treated and untreated samples against a WC ball counterpart at 21 N are shown in Figure 8. Under the current wear conditions, the C3T samples significantly increased the wear resistance of the Zr702 material with a wear factor about two orders of magnitude lower than that of both C2T and Unt samples. Between the C3T samples, a marginally increased performance of the wear resistance was observed in the orders of C3TV, C3TPd, C3TAg, andC3TAu. The SEM observations on the wear tracks of Unt samples revealed a typical adhesive wear feature, as well as the galling and tearing of the Zr materials.

**Figure 9:**
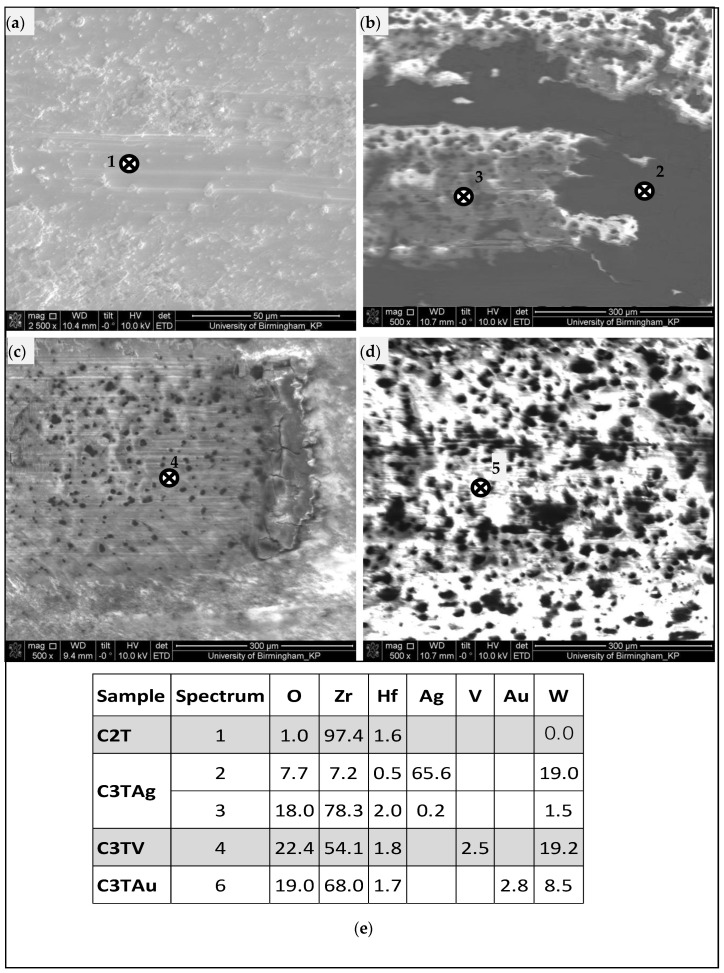
SEM images of wear tracks taken from samples (**a**) C2T; (**b**) C3TAg; (**c**) C3TV; (**d**) C3Tau; and (**e**) EDX spots chemical analysis (wt.%) in the wear tracks as indicated within (**a**–**d**).

The C2T-treated samples experienced severe wear, which was even marginally worse than Unt’s performance. As evidenced in Figure 9a, there is a large number of wear debris in the wear tracks and a few numbers of furrows with great depth. This is because the thin surface oxide layer was easily removed during the reciprocating sliding process, which could then act as the third body to abrasively wear off the relatively soft substrate materials excessively.

The wear tracks of C3T samples showed different morphologies to that of the Unt and C2T samples. Generally, shallow and flat wear tracks (Figure 8b) with few debris and mild furrows were observed, as exemplified in Figure 9b,c for all C3T samples. Moreover, some different features related to the pre-deposited elements were observed on C3T samples. It was found that the wear tracks of C3TAg were partially covered with black patches and a typical morphology is shown in Figure 9b. The EDX analysis in the black patch areas revealed a high content of Ag and W. This implies that, during the sliding process, surface dendrite particles, such as Ag and AgO_2_, protruded from the surface, as described in Section 3.2, and were worn off by the WC ball first, and then compacted between the contacting parts after a series of cycle processes, thus forming a self-lubricating film mainly consisting of soft Ag.

In the meantime, the asperities on the WC ball played a ploughing effect on the exposed ZrO_2_ hard surface, as evidenced in Figure 9b, with spectrum 3 showing high Zr and O. As the hardness of the tribo-pair is similar, HV1328 for WC-CO and HV1350 for ZrO_2_ [18], when silver or silver oxide were worn out, the asperities on the ZrO_2_ surface were ploughed against the WC ball, and the debris were imbedded in the self-lubricating silver rich black patch/film during the sliding cyclic processes, thus reducing the wear damage of these third-body particles to the surface of the C3TAg samples.

Similar, but smaller, self-lubricating patches and light furrows were observed on the C3TAu wear tracks (Figure 9d). However, because of the smaller Ra and Rt values of the C3TAu than the C3TAg sample, reduced asperities of the ZrO_2_ were presented, which led to less wear debris from the WC ball, as supported by the lower W content detected from the black patch areas 5 (C3TAu) than from area 2 (C3TAg).

For the C3TV and C3TPd samples, the formed super-surface oxides of V_2_O_5_ and PdO possess a high hardness over HV3000 [19], which resulted in the wear mechanism differing from that for the C3TAg and C3TAu. At the beginning of the wear, the asperities of the superficial hard oxides cut the WC balls, as evidenced by the high friction coefficient of the tribo-pair (Figure 7), involving C3TV and C3TPd, and by the high content of W in the wear tracks of C3TV (Figure 9e). The cyclic loading would lead to the initiation and growth of fatigue cracks on the superficial oxides, and once the crack propagation developed to a critical degree, flaking off of the superficial oxides appeared. The very thin flakes were ground into small-sized abrasive particles by the relative motion of the tribo pair, and abrasive wear occurred on the exposed ZrO_2_ surface. At this point, the cutting of the WC-Co ball by hard oxides of V_2_O_5_ and PdO was finished, which resulted in the reduced CoF, as shown in Figure 7 after 100 and 400 cycles for the C3TPd and C3TV samples, respectively. As the superficial oxides are of low quantity, as indicated by the low content of V and Pd by EDX analysis for the C3TV and C3TPd samples in Figure 9e, and the underneath ceramic layer of ZrO_2_ is hard and thick enough to resist the abrasive wear and to withstand the normal load applied, the wear rates of these two samples are still very low, compared with the C2T sample, but are about 20, and 80% for the C3TPd and C3TV samples, respectively, higher than the C3TAu and C3TAg samples.

## 4. Discussion

### 4.1. Catalytic Effect of Pre-Deposition Elements on the Growth of the Oxide Layer

By the pre-deposition of Ag, V, Pd, or Au elements, the ceramic conversion process via thermal oxidation for the Zr702 alloy can be effectively promoted, as compared with the samples without pre-deposition (C2T). It can be seen from Figure 1 that, with the pre-deposition of Ag, the thickness of the oxide layer was increased significantly when treated at 550 °C. When further increasing the treatment temperature to 600 °C, the oxide layer thickness was only marginally increased, which differed greatly from the catalytic effect of other pre-deposited elements in this temperature range. This might be related to Ag agglomeration into larger dendrite-like particles (Figure 2) at high temperatures, which may have limited the catalytic effect of Ag on oxide layer growth.

Vanadium pentoxide (V_2_O_5_) is commonly used as the catalyst in producing sulfuric acid [20] in the temperature range of 400–600 °C. The formation of the vanadium pentoxide (V_2_O_5_) identified via the XRD on the Zr702 surface might indicate a similar catalytic effect in promoting the oxidation process for Zr702. Au (111) surfaces, which were detected via the XRD on the C3TAu sample, were reported as effective catalysts in surface reactions, such as selective oxidation and oxidative coupling [21]; hence, they could speed up the oxidation process for Zr702. However, the catalytic effect of Au is not as strong as other elements (Ag, V, and Pd) in the temperature range from 550 °C to 600 °C. Judging by the oxide layer thickness, Ag and V showed the best catalytic effect at 600 °C for 10 h.

### 4.2. Improved Tribological Performance

As reported in Figure 7, untreated Zr702 sample exhibited a high coefficient of friction (CoF) value up to about 0.65, which is consistent with the reported friction coefficient for zirconium alloys [22]. As shown in Figure 7, both the C2T and C3T techniques significantly decreased the coefficient of friction (CoF) of Zr702 alloy. This is due to the in-situ conversion of soft metallic Zr702 into hard ceramic ZrO_2_. It is also noticed from Figure 7 that the CoF for both the C3TV and C3TPd samples fluctuated at the beginning, before it returned to low and stable values after about 400 and 100 cycles for C3TPd and C3TV, respectively. This was caused by the asperities of the superficial hard oxides that cut the WC balls. As the cyclic loading would lead to the superficial layer flaking off, they were ground into small sized abrasive particles by the relative motion of the tribo-pair and abrasive wear occurred on the exposed ZrO_2_ surface, which resulted in the reduced CoF. However, Ouyang, Murakami, and Sasaki [23] reported that the formation of V_2_O_5_ oxide layers could effectively reduce the CoF, and they considered V_2_O_5_ as a solid lubricant, due to its layered structure [24,25]. The conflicting results from this study are related to the different tribo-pair used. In their research, a harder counterpart ball of Si_3_N_4_, rather than a WC-Co ball, was used, and this eliminated the oxides cutting the counterpart balls and, therefore, received a constant low CoF.

The CoF values of Au are the most stable and the lowest in all the C3T-treated samples, mainly because of the relatively low roughness and the lubricant effects of Au [26]. The addition of the catalytic elements effectively reduces the wear rate of the Zr702 samples (Figure 8), with the best performance for the C3TAg and C3TAu samples. This could be explained by the formation of thick and hard surface oxide layer on the top of the C3T-treated surfaces. Owing to the increase of the surface hardness and the nature of the ceramic/metal contact, based on the thicker ceramic layer formed, the severe abrasive and adhesive wear of Zr702 alloy changed to mild abrasive wear. The two orders of magnitude reduction of the wear factor effectively reduced the CoF from 0.65 to <0.30, which clearly demonstrated the effectiveness of the C3T techniques in improving the tribological properties of Zr702 alloy.

## 5. Conclusions

By introducing a pre-deposited catalytic film, the successful promotion of the C2T process, in terms of reduced treatment time, significantly improved the surface hardness and tribological performance of Zr702 alloy. This novel catalytic ceramic conversion treatment (C3T) to Zr702 greatly reduced energy consumption and is an efficient method for real industrial applications. Based on the results presented, the following three conclusions can be made:

1. A new catalytic ceramic conversion treatment (C3T) technique to improve the tribological properties of Zr702 alloy has been developed by adding a thin (300–350 nm) specific catalytic film (Ag, Au, V, or Pd) onto the surface before ceramic conversion treatment (C2T) via thermal oxidation.
2. Compared with conventional C2T, the C3T technique can produce hard, dense, and adherent oxide layers with a thickness at least twice that produced by C2T under the same temperature/time conditions. Ag and V showed a stronger catalytic effect than Pd and Au.
3. The C3T technique can provide two orders of magnitude reduction of wear factor and reduce the coefficient of friction from 0.65 to <0.25. Among the C3T samples, the C3TAg and the C3TAu samples have the highest wear resistance and lowest CoF, mainly due to the self-lubricant formation during the wear processes.

## Figures and Tables

**Table 1:** The chemical composition (wt.%) of Zr 702 alloy.

	Element	Zr + Hf, max	Hf, max	Fe + Cr, max	C, max	O, max
Material						
Zr702		99.2	4.5	0.20	0.05	0.16

**Table 2:** Treatment conditions and sample codes.

Sample Code	Pre-Deposition	Temperature/°C	Time/Hour
C3TAg	Ag	450, 500, 550, 600	10
C3TV	V	450, 500, 550, 600	10
C3TPd	Pd	450, 500, 550, 600	10
C3TAu	Au	450, 500, 550, 600	10
C2T	None	450, 500, 550, 600	10
Unt	-	-	-

## Data Availability

The data presented in this study are available in article.
